# Genome sequencing of fowl cholera vaccine strain *Pasteurella multocida* in Bangladesh

**DOI:** 10.1128/mra.00614-26

**Published:** 2026-06-29

**Authors:** Mahinur Begum Mokta, Md Rezaul Karim, Taposhi Rani Dey, Sharmin Alam, A. N. M. Golam Mohiuddin, Md. Zahangir Hosain, Md. Mostofa Kamal, Mohammed Abdus Samad

**Affiliations:** 1 Livestock Research Institute, Department of Livestock Services 560480, Dhaka, Bangladesh; 2 Animal Health Research Division, Bangladesh Livestock Research Institute 560480, Savar, Dhaka, Bangladesh; 3 Transboundary Animal Disease Research Center, Bangladesh Livestock Research Institute 560480, Savar, Dhaka, Bangladesh; DOE Joint Genome Institute, Berkeley, California, USA

**Keywords:** fowl cholera vaccine, *Pasteurella multocida*, genome sequence

## Abstract

Fowl cholera is a severe infectious disease of domestic and wild avian species caused by *Pasteurella multocida*. In this study, we report the genome sequence of the fowl cholera vaccine strain, BLRI-LRI-FC-vac. The assembled genome was approximately 2,330,753 bp, with 70× sequencing coverage and a genomic G + C content of 40.4%.

## ANNOUNCEMENT

*Pasteurella multocida* is the etiologic agent of fowl cholera, a highly contagious and severe disease of poultry causing septicemia and high mortality, resulting in significant economic losses to the poultry industry worldwide (1). In Bangladesh, about 25%–35% mortality in chickens is caused by FC (2, 3). *P. multocida* is a Gram-negative, non-motile, coccobacillus, capsulated, non-spore-forming bacterium occurring singly, in pairs, or occasionally as chains or filaments belonging to the *Pasteurellaceae* family (4). Here, we announce the genome sequence of *P. multocida* strain isolated from the fowl cholera vaccine for providing valuable information regarding genome structure, sequence identity, MLST profile, and genetic stability. The fowl cholera vaccine strain master seed obtained from the Bacterial Vaccine Production Unit of LRI, Bangladesh, was enriched overnight at 37°C in Luria–Bertani (LB) broth (Oxoid, Thermo Fisher Scientific, UK). Subsequently, a loopful of the enriched culture was streaked onto blood agar base supplemented with 5% sheep blood (Oxoid, Thermo Fisher Scientific, UK) and incubated at 37°C for 24-48 h. Genomic DNA was extracted from a single colony using the QIAamp DNA Mini Kit (Cat. No. 51304, Germany), following the manufacturer’s protocol. Sequencing libraries were then prepared using the Illumina DNA Prep Library Preparation Kit (Illumina, Inc., San Diego, CA, USA). Sequencing was performed with 2 × 150 bp paired-end chemistry on Illumina NextSeq 2000. Sequencing of strain BLRI-LRI-FC-vac generated 2,900,243 reads, with an average read length of 150 bp. FastQC v0.12.1 (5) was used to check the quality of sequence reads and filtered using Fastp v0.24.0 (6), then *de novo* assembled with SPAdes v. 4.0.0 (7), and the post-quality resulting assembly was checked with QUAST v5.3.0 (8). To assess assembly accuracy, genome completeness, and contamination were determined with BUSCO v5.8.0 (9), and average nucleotide identity (ANI) values were determined through the FastANI v1.3 (10) to a reference (accession GCF_002073255.2). Genome annotation of *P. multocida* strain BLR-LRI-FC-vac was performed using the NCBI Prokaryotic Genome Annotation Pipeline (PGAP) v6.9 (11). All analyses were performed using default settings, unless otherwise specified.

The assembled genome of *P. multocida* strain BLRI-LRI-FC-vac comprises 2,330,753 bp spanning over 44 contigs with genome coverage of 70×. The GC content was 40.4%. Assembly quality metrics included L50 values of 6, with corresponding N50 values of 164,952. A summary of genome characteristics is provided in Table 1.

**TABLE 1:** General features of the complete genome sequence of fowl cholera vaccine strain, *P. multocida* strain BLRI-LRI-FC-vac^*a*^

Genomic feature	*P. multocida* BLRI-LRI-FC
BioSample accession	SAMN54763382
GenBank accession	NZ_JBUDMF000000000.1
Assembly accession	GCF_054958685.1
Genome size	2,330,753 bp
GC content	40.4%
MLST	ST 44
Genes (total)	2,230
CDSs (total)	2,172
Genes (coding)	2,144
CDSs (with protein)	2,144
Genes (RNA)	58
rRNAs	2, 1, 1 (5S, 16S, 23S)
Complete rRNAs	2, 1, 1 (5S, 16S, 23S)
tRNAs	50
ncRNAs	4
Pseudo genes (total)	28
CDSs (without protein)	28
Pseudo genes (ambiguous residues)	0 of 28
Pseudo genes (frameshifted)	7 of 28
Pseudo genes (incomplete)	13 of 28
Pseudo genes (internal stop)	11 of 28
Pseudo genes (multiple problems)	2 of 28
BUSCO completeness	98.5
BUSCO contamination	0.1
Genome quality	Good
Average nucleotide identity (ANI)	98.55
N50	164,952
N90	32,710
auN	141,302
L50	6
L90	21

^*a*^ MLST, Multilocus sequence typing (MLST) was determined by analyzing WGS in PubMLST web tools.

## Data Availability

The Whole Genome Shotgun project of the fowl cholera vaccine strain, *P. multocida* BLRI-LRI-FC-vac, has been deposited in GenBank and the NCBI Sequence Read Archive (SRA) under accession SRR38669394. The version described in this paper is version NZ_JBUDMF000000000.1.
